# CARM1 promotes non-small cell lung cancer progression through upregulating CCNE2 expression

**DOI:** 10.18632/aging.103280

**Published:** 2020-06-02

**Authors:** Deqin Wu, Jing He, Wei Zhang, Kai Wang, Shidai Jin, Jun Li, Wen Gao

**Affiliations:** 1 Department of Oncology, The First Affiliated Hospital of Nanjing Medical University, Nanjing 210029, China; 2 Department of Pharmacy, The First Affiliated Hospital of Nanjing Medical University, Nanjing 210029, China; 3 Department of Radiology, The First Affiliated Hospital of Nanjing Medical University, Nanjing 210029, China; 4 Department of Radiotherapy Oncology, Nanjing Benq Medical Center, Nanjing 210019, China

**Keywords:** CARM1, CCNE2, proliferation, NSCLC, methylation

## Abstract

The underlying molecular mechanisms of tumorigenesis and progression of non-small cell lung cancer (NSCLC) are not yet fully elucidated. In the present study, *in* *vitro* functional dissections suggest that siRNA-mediated silencing of CCNE2 profoundly attenuated the proliferative and colony-formative abilities of NSCLC PC9 and HCC827 cells, while forced overexpression of CCNE2 significantly strengthened the proliferative and colony-formative capabilities of these cells. Intriguingly, by ChIP and luciferase reporter gene assays, we observed that CARM1 is recruited to the promoter regions of CCNE2 gene and acts as a transcriptional activator. Mechanically, the asymmetric di-methylation of H3R17me2a and H3R26me2a, as the catalytic substrates of CARM1, were highly enriched at the core promoter regions of CCNE2 gene, thereby activating the expression of CCNE2. *In vitro* and *in vivo* rescue experiments demonstrated that restoration of CCNE2 expression significantly abolished the CARM1 shRNA-mediated inhibition of cell proliferation, indicating that the oncogenic function of CARM1, at least partially, depended on the activation of CCNE2. Inhibition of CARM1 enzymatic activity could significantly repress CCNE2 expression in NSCLC cells. In addition, the expression of CARM1 was significantly elevated and positively correlated with CCNE2 levels in 20 cases of NSCLC patients. Both CARM1 and CCNE2 are highly associated with shorter 10-year overall survival of at a large cohort of 461 cases of NSCLC patients from the Kaplan-Meier plotter database. To summarize, these findings provide compelling evidence that CARM1 could promote NSCLC progression via activation of CCNE2, paving the way for future therapeutic strategies in NSCLC.

## INTRODUCTION

Lung cancer is the leading cause of cancer-related deaths worldwide, and more than 80% of the lung cancer cases are non-small cell lung cancer (NSCLC) [1, 2]. Unfortunately, the morbidity and mortality rates of NSCLC are increasing annually because of the high prevalence of cigarette smoking, serious air pollution, environmental deterioration and other external factors. Despite advances in surgical technique and improvements in adjuvant radiotherapy and chemotherapy, treatment of NSCLC is remained as an important and nontrivial challenge in clinical oncology. Treatment failure is still inevitable in most cases of NSCLC due to the high risk for metastasis, chemo/radio-resistance and recurrence. Up until now, the underlying molecular mechanisms of tumorigenesis and progression are not yet well defined [3, 4]. Therefore, there is an urgent need to characterize novel key regulators controlling tumorigenesis and progression of NSCLC, although this is still at the preliminary exploratory stage.

In eukaryotic cells, the progression of cell cycle is controlled by a conserved family of cyclin-dependent kinases (CDKs), including Cyclin E (CCNE) [5, 6]. Cyclin E2 (CCNE2) is identified as the second member of E-type CDKs, which contributes to the G_1_/S phase transition, cell proliferation, tumorigenesis and cancer progression [7]. CCNE2 overexpression is frequently observed in AML, breast cancer, lung cancer and gastric cancer [8]. Despite rigorous effort, the underlying mechanisms of CCNE2 involved in the tumorigenesis and cancer progression are still largely unknown.

Coactivator-associated arginine methyltransferase 1 (CARM1), also known as PRMT4, is an important member of protein arginine methyltransferase (PRMT) family [9]. As an epigenetic regulator, CARM1 exerts its transcriptional control by asymmetrically di-methylating arginine residues on histones, transcription factors, RNA polymerase II and other regulators [10]. Multiple lines of evidence suggest that CARM1 plays crucial roles in modulating a variety of cellular processes, such as transcription activation [11], RNA processing [12], tumorigenesis and cancer progression [13], cell growth/differentiation [14] and apoptosis [15]. Alteration of CARM1, mostly upregulation, was frequently reported in various types of human cancers, including breast cancer, prostate cancer and colorectal cancer, which appears to promote cancer initiation, progression and metastasis. [13] CARM1 elevation not only modulates the activity of cancer-related signaling pathways, but also creates a favorable microenvironment for tumorigenesis and cancer progression.

In this study, we aim to elucidate the potential roles of CARM1 and related target genes in NSCLC cancer progression. Our study showed that CARM1 is recruited to the promoter regions of CCNE2 gene and could promote NSCLC progression via activation of CCNE2 expression. These discoveries help us better understand the regulation of cancer progression and provide a novel therapeutic strategy for NSCLC.

## RESULTS

### CCNE2 promotes NSCLC cell proliferation *in vitro*

**Figure 1:**
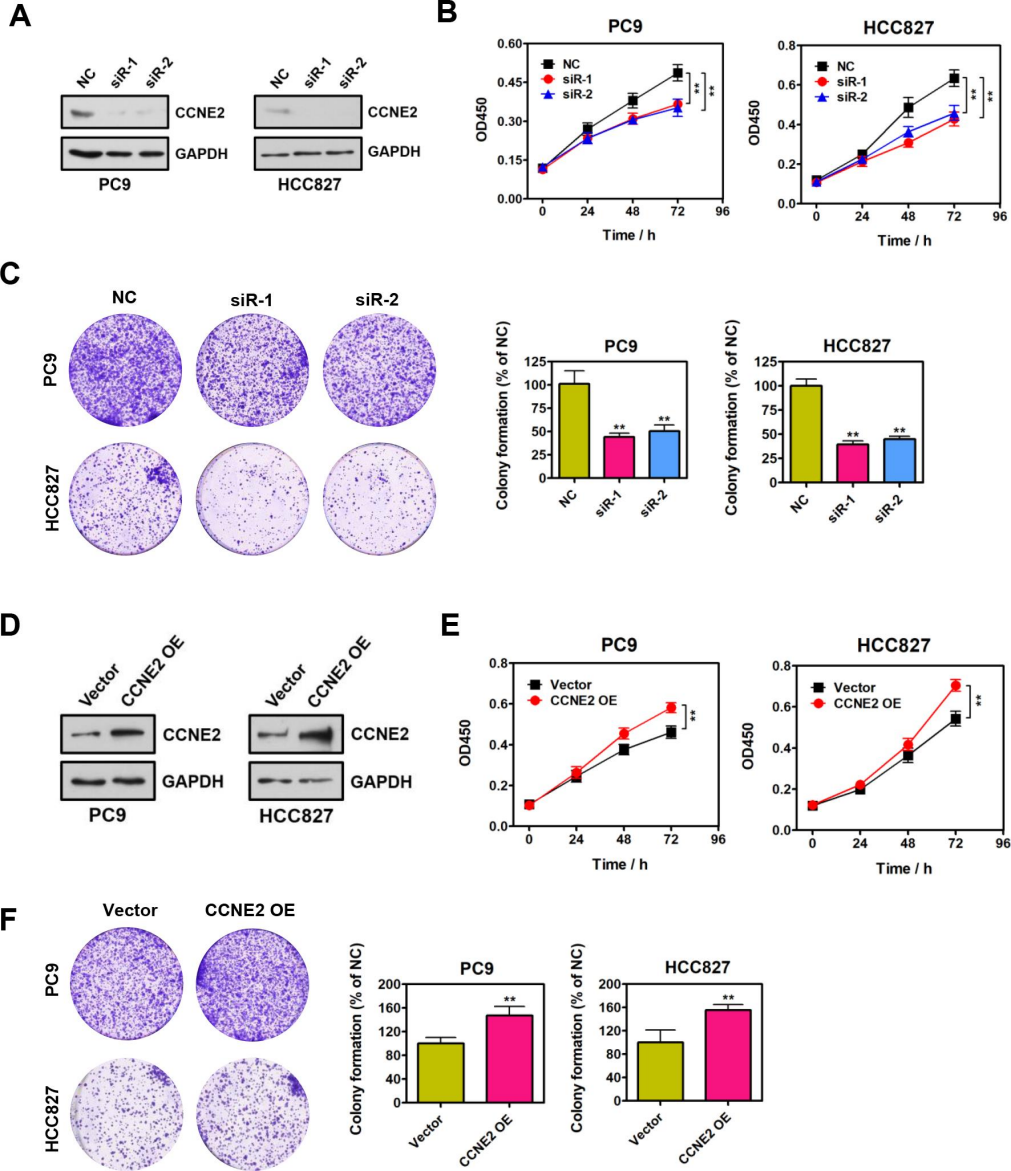
**CCNE2 promotes NSCLC cell proliferation *in vitro.*** (**A**) The knockdown of CCNE2 by siRNAs in PC9 and HCC827 cells was verified by Western blot. GAPDH was used as loading control. (**B**) Cell proliferation abilities of CCNE2-depleted PC9 and HCC827 cells were assessed by CCK-8 assays. The data were presented as means ± SDs of three independent experiments; **P < 0.01. (**C**) Colony-formative abilities of CCNE2-depleted PC9 and HCC827 cells were determined by colony-formation assays. Right panel, the relative colony-formative abilities (% of NC) were quantified. The data were shown as means ± SDs of three independent experiments; **P < 0.01. (**D**) Overexpression of CCNE2 in PC9 and HCC827 cells was examined by Western blot. GAPDH was used as loading control. (**E**) Cell proliferation capacities of CCNE2-overexpressed PC9 and HCC827 cells were assessed by CCK-8 assays. The data were presented as means ± SDs of three independent experiments; **P < 0.01. (**F**) Colony-formative abilities of CCNE2-overexpressed PC9 and HCC827 cells were determined by colony-formation assays. Right panel, the relative colony-formative abilities (% of NC) were quantified. The data were shown as means ± SDs of three independent experiments; **P < 0.01.

We functionally dissected the roles of CCNE2 for the proliferative phenotype of PC9 and HCC827 cells. Specific siRNAs against CCNE2 and negative control (NC) were *instantaneously* transfected into PC9 and HCC827 cells. Reduced protein levels of endogenous CCNE2 was confirmed by Western blot analysis, as shown in Figure 1A. Cell proliferation was assessed by CCK-8 assays. According to the CCK-8 results, the proliferative ability of PC9 and HCC827 cells with CCNE2 knockdown was significantly lower than that of NC cells (Figure 1B; **P < 0.01). Colony-formation results suggest that depletion of CCNE2 significantly inhibited the colony-formative ability of PC9 and HCC827 cells (Figure 1C; **P < 0.01). To further determine whether CCNE2 is required for the proliferation of these NSCLC cells, we overexpressed CCNE2 by transfecting the recombinant pcDNA3.1-CCNE2 plasmid into PC9 and HCC827 cells (Figure 1D). As shown in Figure1E, 1F, the proliferative and colony-formative capabilities of PC9 and HCC827 cells were remarkably higher than that of control cells. In conclusion, these results document that CCNE2 could promote the proliferation and colony-formation of NSCLC cells *in vitro* (**P < 0.01 & **P < 0.01), supporting the tumorigenic role of CCNE2 in NSCLC.

### CARM1 is a positive regulator of CCNE2 gene in NSCLC cells

**Figure 2:**
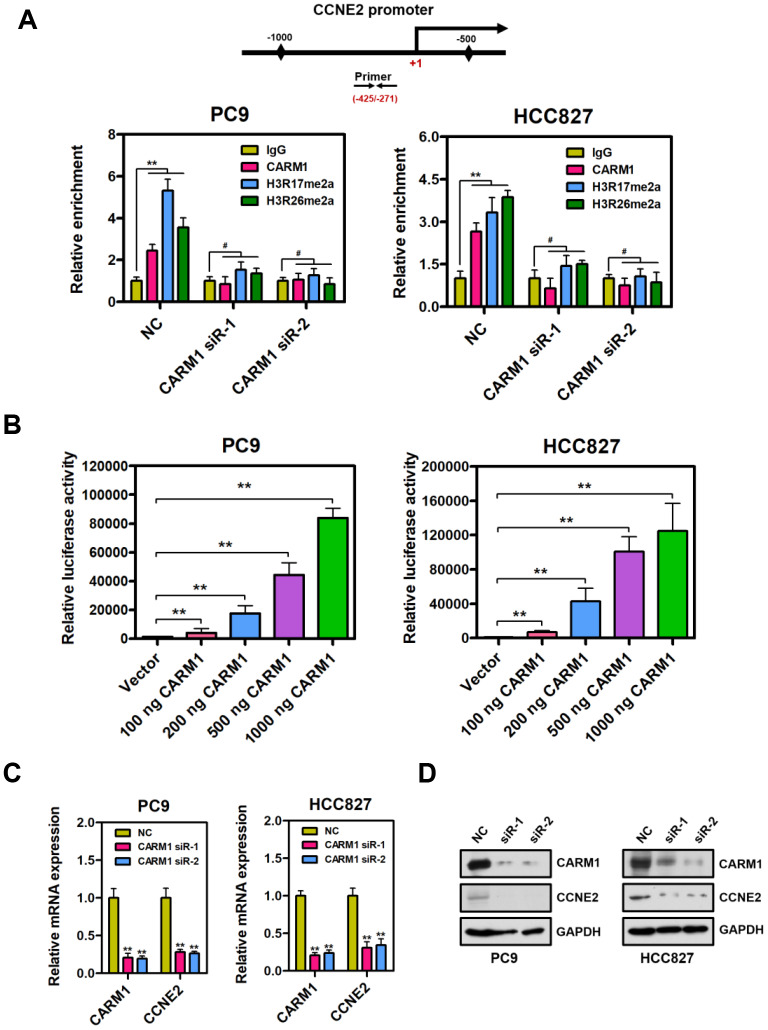
**CARM1 is a positive regulator of CCNE2 gene in NSCLC cells.** (**A**) ChIP analysis of human CCNE2 promoter by antibodies against CARM1, H3R17me2a, H3R26me2a or IgG in NC or CARM1-silenced PC9 and HCC827 cells. Relative enrichment of CARM1, H3R17me2a and H3R26me2a marks on the promoter regions was analyzed by real-time PCR assays. The data were presented as means ± SDs of three independent experiments; **P < 0.01, ^#^*P* > 0.05. (**B**) The luciferase activity of CCNE2 promoter reporter was significantly increased when CARM1 (100 ng, 200 ng, 500 ng and 1000 ng) was transfected into PC9 and HCC827 cells. The CCNE2 promoter reporter luciferase activity was normalized to beta-galactosidase activity. The data were shown as means ± SDs of three independent experiments; **P < 0.01. (**C**) The mRNA levels of CCNE2 was downregulated in CARM1-depleted PC9 and HCC827 cells by Real-time PCR assays. β-actin was used as an internal control. The data were presented as means ± SDs of three independent experiments; **P < 0.01. (**D**) The protein levels of CCNE2 was downregulated in CARM1-depleted PC9 and HCC827 cells by Western blot. GAPDH was used as loading control.

By chromatin immunoprecipitation (ChIP) assays, we observed that CARM1 was enriched at the promoter region of CCNE2 gene in PC9 and HCC827 cells (Figure 2A; **P < 0.01). It is well known that CARM1 is an important transcriptional co-activator and exerts its transcriptional activation through asymmetrical di-methylation of arginine residues. Intriguingly, we extended this observation and found that CARM1-mediated histone marks H3R17me2a and H3R26me2a were also obviously accumulated at the CARM1-enriched promoter region of CCNE2 gene in PC9 and HCC827 cells. Notably, CARM1 and its two modifications (H3R17me2a and H3R26me2a) were almost undetectable at the promoter region of CCNE2 gene in CARM1-depleted PC9 and HCC827 cells. It is worth pointing out that, by luciferase reporter gene assays, CARM1 could directly contribute to activate CCNE2 promoter reporter in PC9 and HCC827 cells. (Figure 2B; **P < 0.01). The luciferase activity of CCNE2 promoter reporter was significantly increased when CARM1 (100 ng, 200 ng, 500 ng and 1000 ng) was transfected into PC9 and HCC827 cells in a concentration-dependent manner.

Real-time PCR analysis showed that CCNE2 was downregulated in PC9 and HCC827 cells when CARM1 was interfered by shRNA (Figure 2C; β-actin as internal control, **P < 0.01). It should be mentioned that a similar real-time PCR result was obtained when GAPDH was used as an internal control (Supplementary Figure 1; **P < 0.01). To further confirm these results, we examined the protein expression of CCNE2 in CARM1-depleted PC9 and HCC827 cells by Western blot assays. As a result, the protein expression of CCNE2 was also remarkably reduced in CARM1-depleted PC9 and HCC827 cells (Figure 2D). To summarize, these data indicate that CARM1 is recruited to the promoter region of CCNE2 gene and acts as a transcriptional activator through asymmetrically di-methylating H3R17 and H3R26 in NSCLC cells.

### CARM1 promotes NSCLC cell proliferation *in vitro*

**Figure 3:**
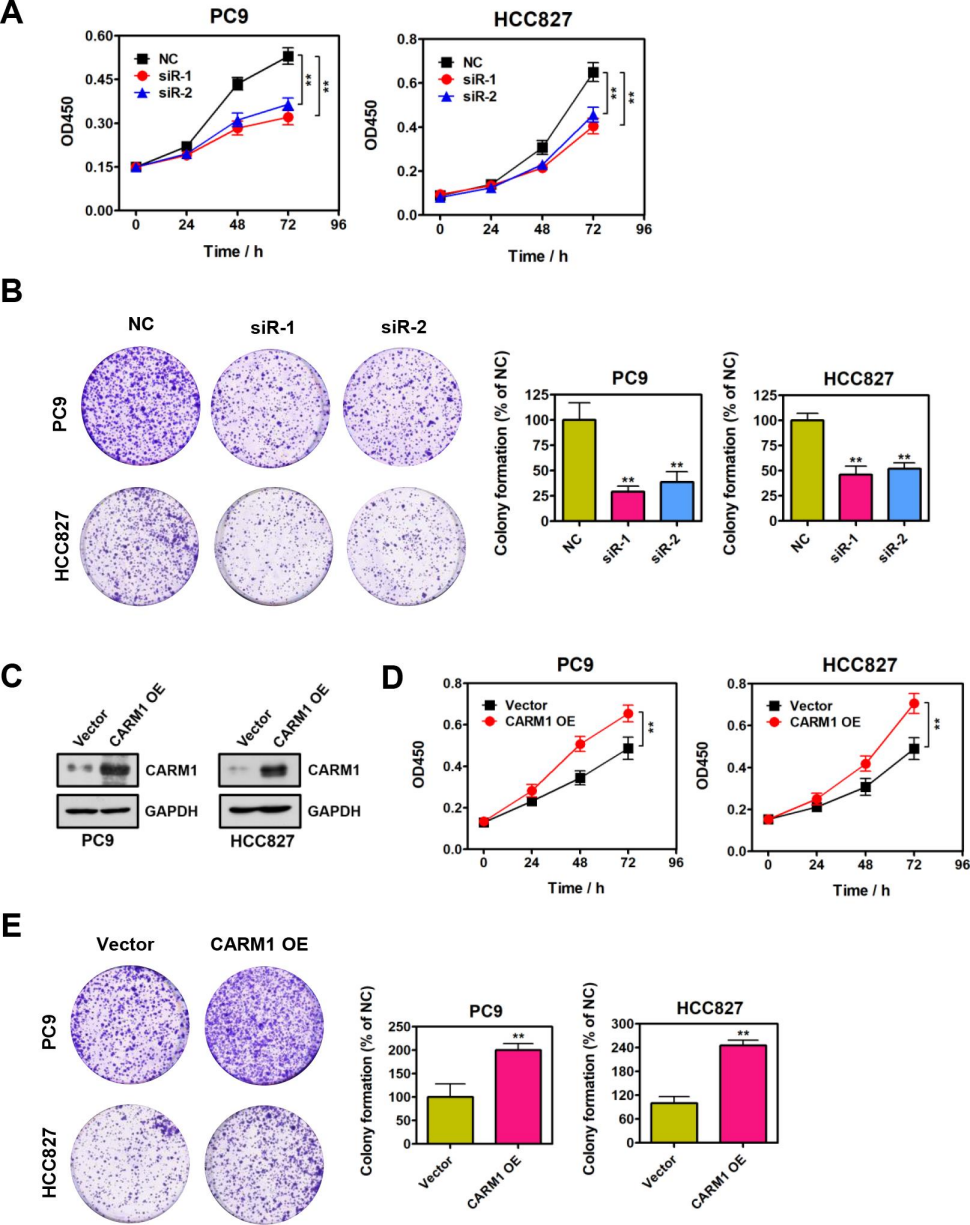
**CARM1 promotes NSCLC cell proliferation *in vitro.*** (**A**) Cell proliferation abilities of CARM1-depleted PC9 and HCC827 cells were assessed by CCK-8 assays. The data were presented as means ± SDs of three independent experiments; **P < 0.01. (**B**) Colony-formative abilities of CARM1-depleted PC9 and HCC827 cells were determined by colony-formation assays. Right panel, the relative colony-formative abilities (% of NC) were quantified. The data were shown as means ± SDs of three independent experiments; **P < 0.01. (**C**) Overexpression of CARM1 in PC9 and HCC827 cells was examined by Western blot. GAPDH was used as loading control. (**D**) Cell proliferative abilities of CARM1-overexpressed PC9 and HCC827 cells were assessed by CCK-8 assays. The data were presented as means ± SDs of three independent experiments; **P < 0.01. (**E**) Colony-formative abilities of CARM1-overexpressed PC9 and HCC827 cells were determined by colony-formation assays. Right panel, the relative colony-formative abilities (% of NC) were quantified. The data were shown as means ± SDs of three independent experiments; **P < 0.01.

To explore the functional role of CARM1 in NSCLC, we evaluated the effect of CARM1 knockdown on the proliferation of PC9 and HCC827 cells. Noticeably, as shown in Figure 3A, the proliferation ability of PC9 and HCC827 cells were substantially reduced when CARM1 was silenced by siRNAs (**P < 0.01). Similarly, colony-formation results strongly suggested that depletion of CARM1 significantly attenuated the colony-formation ability of PC9 and HCC827 cells (Figure 3B; **P < 0.01). In contrast, the proliferative and colony-formative abilities of PC9 and HCC827 cells were notably enhanced when CARM1 was overexpressed by transfecting the recombinant pcDNA3.1-CARM1 plasmid into PC9 and HCC827 cells (Figure 3C–3E). In conclusion, these observations altogether suggest that CARM1 could promote NSCLC cell proliferation and colony-formation *in vitro*, demonstrating the tumor-promoting role of CARM1 in NSCLC.

### Inhibition of CARM1 enzymatic activity represses CCNE2 expression in NSCLC cells

**Figure 4:**
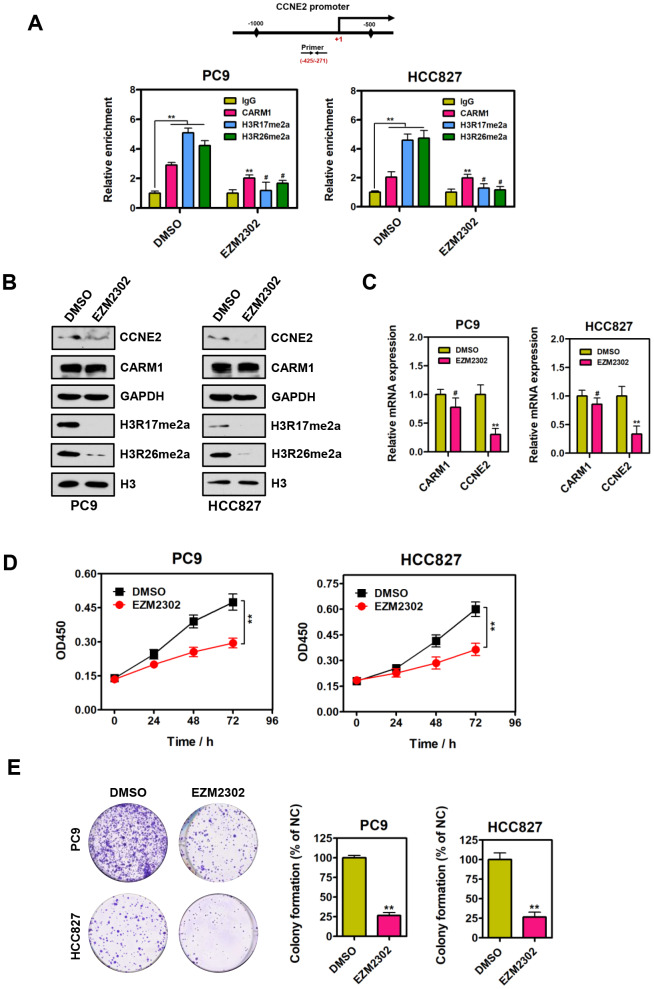
**Inhibition of CARM1 enzymatic activity represses CCNE2 expression in NSCLC cells.** (**A**) ChIP analysis of human CCNE2 promoter by antibodies against CARM1, H3R17me2a, H3R26me2a or IgG in DMSO or EZM2302-treated (10 nM) PC9 and HCC827 cells. Relative enrichment of CARM1, H3R17me2a and H3R26me2a marks on the promoter regions was analyzed by real-time PCR assays. The data were presented as means ± SDs of three independent experiments; **P < 0.01, ^#^*P* > 0.05. (**B**) The protein levels of CCNE2, H3R17me2a and H3R26me2a were downregulated in EZM2302-treated (10 nM) PC9 and HCC827 cells by Western blot. GAPDH or histone H3 were used as loading controls. (**C**) The mRNA levels of CCNE2 was downregulated in EZM2302-treated (10 nM) PC9 and HCC827 cells by Real-time PCR assays. β-actin was used as an internal control. The data were shown as means ± SDs of three independent experiments; **P < 0.01. (**D**) Cell proliferation abilities of DMSO or EZM2302-treated (10 nM) PC9 and HCC827 cells were assessed by CCK-8 assays. The data were presented as means ± SDs of three independent experiments; **P < 0.01. (**E**) Colony-formative abilities of DMSO or EZM2302-treated (10 nM) PC9 and HCC827 cells were determined by colony-formation assays. Right panel, the relative colony-formative abilities (% of NC) were quantified. The data were shown as means ± SDs of three independent experiments; **P < 0.01.

Since CARM1 is an important arginine methyltransferase, it would be of interest to determine whether the enzymatic activity of CARM1 plays a central role in the regulation of CCNE2 in NSCLC cells. EZM2302 (GSK3359088) is a potent and selective inhibitor of CARM1 enzymatic activity. Therefore, we performed ChIP assays using antibodies against IgG, CARM1, H3R17me2a and H3R26me2a in PC9 and HCC827 cells upon EZM2302 treatment. As shown in Figure 4A, we observed that the enrichment of H3R17me2a and H3R26me2a (catalyzed by CARM1) at the promoter region of CCNE2 gene was dramatically reduced when NSCLC PC9 and HCC827 cells were treated with EZM2302. Furthermore, Western blot and quantitative real-time PCR were performed to determine both the protein and mRNA levels of CCNE2 in NSCLC PC9 and HCC827 cells treated with EZM2302. As expected, the western blot analysis results showed that EZM2302 inhibited the enzymatic activity of CARM1, leading to significantly reduced H3R17me2a and H3R26me2a levels. The protein and mRNA expression of CCNE2 was consistently reduced, although the expression of CARM1 was not changed significantly (Figure 4B, 4C). To assess the inhibitory effect of this inhibitor on NSCLC cells, we treated NSCLC PC9 and HCC827 cells with EZM2302. As expected, we found that inhibition of CARM1 enzymatic activity by EZM2302 significantly inhibited the proliferative and colony-forming abilities of NSCLC PC9 and HCC827 cells (Figure 4D and 4E; **P < 0.01). These results are consistent with those obtained in the CARM1 knockdown experiments, indicating that the inhibition of CARM1 enzymatic activity results in the repression of CCNE2 expression in NSCLC cells and subsequently inhibits NSCLC progression.

### Restoration of CCNE2 expression abrogated the proliferation inhibition caused by CARM1 knockdown

**Figure 5:**
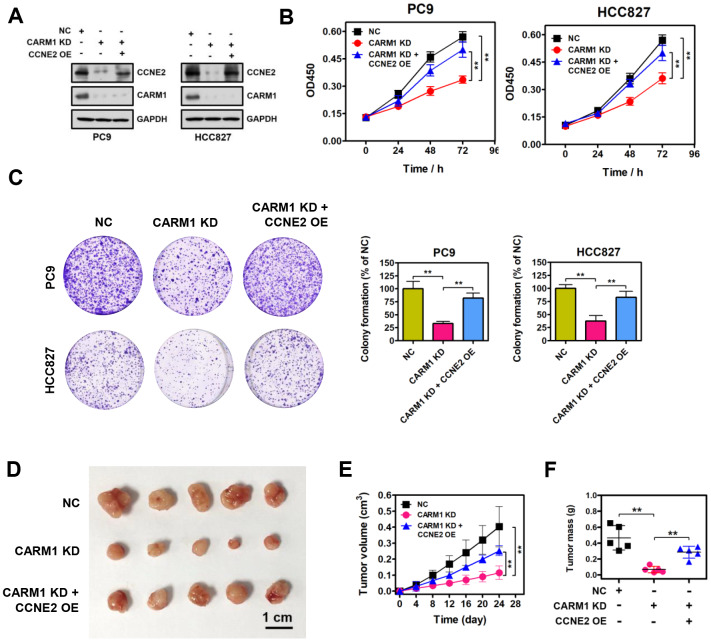
**Restoration of CCNE2 expression abrogates the proliferation inhibition caused by CARM1 knockdown.** (**A**) The restoration of CCNE2 in CARM1-depleted PC9 and HCC827 cells was verified by Western blot. GAPDH was used as loading control. (**B**) Cell proliferation capacities of NC, CARM1 KD (CARM1 shRNA) and CARM1 KD + CCNE2 OE (CCNE2 overexpression)-treated PC9 and HCC827 cells were determined by CCK-8 assays. The data were presented as means ± SDs of three independent experiments; **P < 0.01. (**C**) Colony-formative abilities of NC, CARM1 KD and CARM1 KD + CCNE2 OE-treated PC9 and HCC827 cells were determined by colony-formation assays. Right panel, the relative colony-formative abilities (% of NC) were quantified. The data were shown as means ± SDs of three independent experiments; **P < 0.01. (**D**) NC, CARM1 KD and CARM1 KD + CCNE2 OE-treated PC9 cells were subcutaneously injected into the flank of nude mice. Representative images of xenograft tumors excised from mice. (**E**) Tumor growth curves of NC, CARM1 KD and CARM1 KD + CCNE2 OE-treated PC9 cells in nude mice; n =5, **P < 0.01. (**F**) Tumor weights of NC, CARM1 KD and CARM1 KD + CCNE2 OE-treated PC9 xenograft tumors excised from mice; n =5, **P < 0.01.

To determine whether CARM1 promotes cell proliferation of NSCLC cells through activating CCNE2 expression, we restored CCNE2 expression in CARM1-depleted PC9 and HCC827 cells. Enforced restoration of CCNE2 expression in CARM1-depleted PC9 and HCC827 cells was verified by Western blot, as shown in Figure 5A. Strikingly, CCK-8 and colony-formation rescue experiments demonstrated that restoration of CCNE2 expression dramatically impeded the reduction in cell proliferation and colony-formation of PC9 and HCC827 cells mediated by CARM1 knockdown, respectively (Figure 5B, 5C; **P < 0.01). Furthermore, *in vivo* experiments suggested that xenograft tumors of CARM1-knockdown PC9 cells grew significantly slower than the control PC9 tumors. However, enforced restoration of CCNE2 expression dramatically abolished the growth inhibition of PC9 cells mediated by CARM1 knockdown (Figure 5D–5F; **P < 0.01), demonstrating the oncogenic function of CARM1, at least partially, depended on upregulating the expression of CCNE2.

### CARM1 is elevated in NSCLC patients and positively correlated with CCNE2 levels

**Figure 6:**
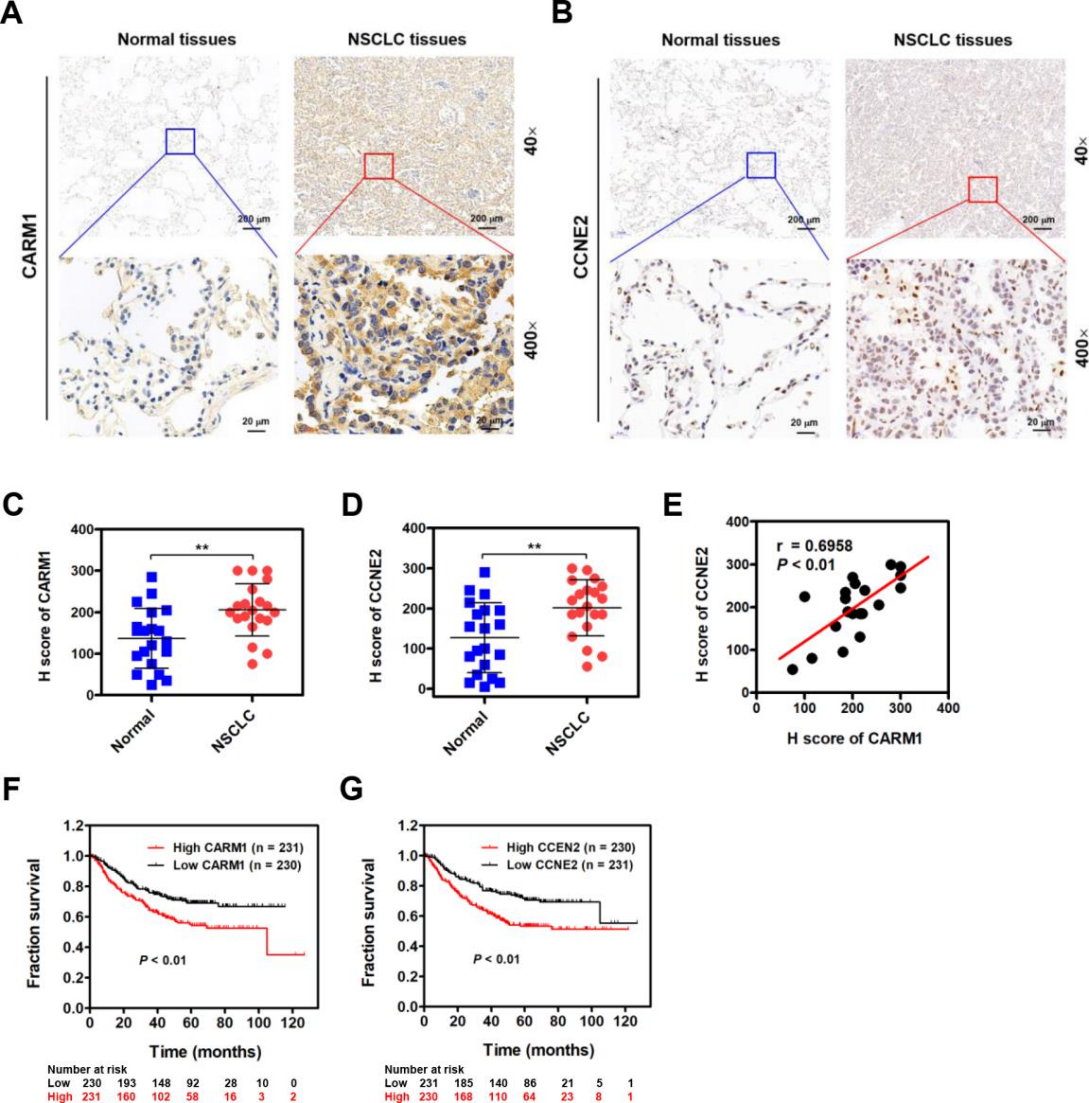
**CARM1 is upregulated in NSCLC patients and positively correlated with CCNE2 levels.** (**A**) Representative images of IHC staining of CARM1 in 20 cases of NSCLC patients (Tumor) and their adjacent non-tumor tissues (Normal). (**B**) Representative images of IHC staining of CCNE2 in 20 cases of NSCLC patients (Tumor) and their adjacent non-tumor tissues (Normal). (**C**) H score of CARM1 expression in 20 cases of NSCLC tumor tissues and their adjacent non-tumor tissues. **P < 0.01. (**D**) H score of CCNE2 expression in 20 cases of NSCLC tumor tissues and their adjacent non-tumor tissues. **P < 0.01. (**E**) Pearson correlation analysis was performed to examine the correlation between CARM1 and CCNE2 expression in 20 cases of NSCLC patients (n = 20; r = 0.6958; *P* < 0.01). (**F**) Analysis of data from the Kaplan-Meier plotter database suggested that high expression of CARM1 (Cutoff value: 262) was associated with shorter 10-year overall survival of NSCLC (lung adenocarcinoma, n = 461) patients. *P* < 0.01. (**G**) Analysis of data from the Kaplan-Meier plotter database suggested that high expression of CCNE2 (Cutoff value: 228) was associated with shorter 10-year overall survival of NSCLC (adenocarcinoma) patients. *P* < 0.01.

Given the role of CARM1 in promoting progression of NSCLC, we therefore examined whether CARM1 is elevated in NSCLC patients by IHC staining. As shown in Figure 6A, CARM1 was immunocytochemically distributed in both cytoplasm and nuclei of tumor cells. Unlike CARM1, CCNE2 was preferentially in the nuclei of tumor cells (Figure 6B). By H score analysis, the expression of both CARM1 and CCNE2 were profoundly elevated in the NSCLC tumor tissues when compared with that in the adjacent non-tumor tissues (Figure 6C, 6D; n =20, **P < 0.01). Given the fact that CCNE2 is stringently regulated by CARM1, we speculate that the expression of CCNE2 probably be related with CARM1 in NSCLC patients. Interestingly, the spearman’s rank correlation analysis revealed that the expression levels of CARM1 and CCNE2 were positively correlated in 20 cases of NSCLC patients (Figure 6E; r = 0.6958, *P* < 0.01). Lastly, we also investigated whether the overexpression of CARM1 or CCNE2 is associated with the overall survival in a large cohort 461 cases of NSCLC (lung adenocarcinoma) patients from the Kaplan-Meier plotter database (https://www.kmplot.com). The median value was selected as the “cutoff” value separating two groups of NSCLC patients with high and low CARM1 or CCNE2 scores. Notably, high expression of CARM1 (Figure 6F; Cutoff value was 262; *P* < 0.01) or CCNE2 (Figure 6G; Cutoff value was 228; *P* < 0.01) was highly associated with shorter 10-year overall survival of NSCLC (adenocarcinoma) patients, implying the oncogenic roles of CARM1 and CCNE2 in promoting the progression of NSCLC.

## DISCUSSION

Methylation of arginine (Arg) residues is a widespread post-translational modification (PTM) involved in a variety of biological processes [16, 17]. The methylation of Arg residues of protein substrates is mainly catalyzed by a family of protein arginine methyltransferases (PRMTs), which includes PRMT4/CARM1. CARM1 exerts its multiple function in the regulation of diverse cellular processes, such as cell proliferation, cell cycle progression and mRNA splicing, through methylating histones, RNA polymerase II and other epigenetic regulators (CREBBP, p300, etc.) [13]. As a transcriptional co-activator, CARM1 is generally correlated with transcriptional activation by catalyzing the asymmetric di-methylation of Arginine 17 and 26 on histone H3 (H3R17me2a and H3R26me2a).

Emerging reports suggest oncogenic functions of CARM1 in human cancer. Messaoudi et al. and Frietze et al. demonstrated that CARM1 could promote cellular proliferation through activating CCNE1 or E2F1 [18, 19]. Wang et al. suggested that CARM1 could promote breast cancer progression and metastasis through methylating chromatin remodeling factor BAF155 at R1064 [20]. However, Dhaheri et al. reported that CARM1 could significantly inhibit estrogen dependent breast cancer cell proliferation through regulating cell cycle [21]. Wang et al. found that overexpression of wild-type CARM1 can significantly reduce the proliferative activity of pancreatic ductal adenocarcinoma cells [22]. In NSCLC, Elakoum et al. showed that CARM1 was elevated in NSCLC cell lines (A549 and H1299) and depletion of CARM1 by siRNAs significantly reduced tumorigenic growth [23]. In this study, we found that the expression of CARM1 was elevated in NSCLC. *In vitro* experiments suggested that silencing of CARM1 could reduce the proliferative activity of NSCLC cells, suggesting the oncogenic functions of CARM1 in NSCLC. Taken together, it is reasonable to conclude that CARM1 functions as either tumor-promoting or anti-proliferative functions, suggesting that the actions of CARM1 might depend on the cellular context and tumor type.

CARM1 asymmetrically di-methylates promoter histones H3R17 and H3R26, and triggers transcriptional activation of cell cycle regulatory and transcriptional factor genes, such as Cyclin E1 (CCNE1) [19], E2F1 [18] and CDKN1A [24] etc. However, to our knowledge, there are no previous reports probing the potential transcriptional targets of CARM1 in NSCLC. Here, we identified Cyclin E2 (CCNE2) is a downstream target gene of CARM1 in NSCLC. Mechanically, CARM1 is recruited to the promoter region of CCNE2 gene and acts as a transcriptional activator through asymmetrically di-methylating H3R17 and H3R26 in NSCLC cells.

CCNE2 is almost undetectable in normal breast cells, and it is significantly upregulated in breast cancer cells [25, 26]. Xie et al. reported that CCNE2 is increased in 308 ovarian cancer samples and high expression of CCNE2 is associated with poor overall survival [27]. In prostate cancer, CCNE2 was proved to be upregulated in patients with prostate cancer and acted as a tumor-promoting protein [28]. In bladder cancer, Matsushita et al. showed that the expression of CCNE1/2 were significantly elevated in 60 specimens compared with 22 normal specimens, and high CCNE1/2 expression associated with lower overall survival probabilities [29]. In NSCLC, Chen et al. found that silencing of CCNE2 could also notably inhibit the proliferative, migrative and invasive activities of NSCLC cells [30]. Collectively, the overexpression of CCNE2 in tumor may promote tumorigenesis and cancer progression through various mechanisms, including increased proliferation, migration and invasion abilities. It is reasonable to conclude that CCNE2 is preferentially expressed in more proliferating cells. However, the underlying mechanisms of how CCNE2 contributes to tumorigenesis and cancer progression is not clear and remains to be elucidated. Similar to previously published results, we observed that CCNE2 upregulation of NSCLC is positively correlated with CARM1 in NSCLC patients. Through the Kaplan-Meier plotter database, we found that high expression of CARM1 or CCNE2 was highly associated with shorter 10-year overall survival of NSCLC (adenocarcinoma) patients, implying the oncogenic roles of CARM1 and CCNE2 in promoting the progression of NSCLC. However, we admit that the survival data of CARM1 and CCNE2 in NSCLC patients using Kaplan-Meier plotter database is somewhat limited because the exact number of inclusive NSCLC patients with TNM stage, grade, gender and the granular clinical or pathological/molecular (EGFR mutations) data is unavailable. CCNE2 siRNA-mediated depletion substantially reduced the proliferative and colony-formative capabilities of NSCLC cells. The *in vitro* and *in vivo* rescue experiments demonstrated that restoration of CCNE2 expression significantly impeded the reduction in cell growth mediated by CARM1 shRNA, indicating that the oncogenic function of CARM1 at least partially depended on activating CCNE2. In conclusion, we identified CARM1 is an important positive regulator of the CCNE2 gene in NSCLC cells. However, one major limitation of this study is the relatively low number of NSCLC patients. Further studies on large cohorts of patients are needed to validate the CARM1-CCNE2 regulatory axis in NSCLC patients. In addition, further *in vivo* studies with a large number of animals, in combination with CARM1 inhibitor, should be optimally designed to validate the CARM1-CCNE2 regulatory axis in xenograft models.

Previous studies have emphasized the importance of CARM1 in resistance to chemotherapy through methylating RNA polymerase II mediator complex subunit 12 (MED12), resulting in highly aggressive breast cancer that insensitive to drugs [31]. It should be mentioned that NSCLC models PC9 and HCC827 cells were epidermal growth factor receptor (EGFR)-mutated, which can survive treatment with EGFR-tyrosine kinase inhibitors (TKIs) until they eventually acquire treatment-resistance. On the basis of the results of this study, we speculate that there seems be to be some relationship between CARM1 and NSCLC resistance to chemotherapy. Therefore, elucidating the roles of CARM1 in regulating the resistance to chemotherapy treatment in NSCLC is an importance issue for future studies.

To conclude, our study demonstrated that CARM1 is significantly elevated in NSCLC, which exerts its oncogenic function through activating CCNE2. Moreover, high expressions of CARM1 and CCNE2 were positively correlated and associated with a poor overall survival of NSCLC patients. Our study not only provides a better understanding of the roles of CARM1-CCNE2 regulatory axis in NSCLC, but also represents promising therapeutic strategies for the treatment of NSCLC.

## MATERIALS AND METHODS

### Patient samples

A total of 20 cases of formalin-fixed and paraffin-embedded NSCLC specimens (including tumor and surrounding non-cancerous tissues) were collected from surgical operation at the First Affiliated Hospital of Nanjing Medical University. This study was approved by the Ethics Committee of the First Affiliated Hospital of Nanjing Medical University. Written informed consents were obtained from all patients who provided samples. For survival analysis, a large cohort 461 cases of NSCLC patients were obtained from the Kaplan-Meier plotter database (https://www.kmplot.com). The median value was selected as the “cutoff” value separating two groups of NSCLC patients with high and low CARM1 or CCNE2 levels. The Affy ID of CARM1 is 212512_s_at; Cutoff value used in analysis is 262; Expression range of the probe is from 64 to 4022. The Affy ID of CCNE2 is 205034_at; Cutoff value used in analysis is 228; Expression range of the probe is from 19 to 3893.

### Cell culture, transfection and EZM2302 treatment

Human NSCLC cell lines PC9 and HCC827 were cultured with Dulbecco’s Modified Eagle’s Medium (DMEM; Gibco) containing 10% (v/v) fetal bovine serum (FBS; Gibco) and 1% (v/v) penicillin/streptomycin under 5% CO_2_ at 37°C. HEK293T cells were cultured with Dulbecco’s Modified Eagle’s Medium (DMEM; Gibco) containing 10% (v/v) fetal bovine serum (FBS; Gibco) and 1% (v/v) penicillin/streptomycin under 5% CO_2_ at 37°C. For transient transfection, about 60% ~ 70% confluence, cells were transfected with siRNAs or plasmids using Lipofectamine 2000 (Life Technologies) according to the manufacturer’s instructions. The transfected cells were cultured for 48 h under 5% CO_2_ at 37°C before being used in subsequent experiments. CARM1 inhibitor-EZM2302 (MCE; HY-111109) was dissolved in dimethyl sulfoxide (DMSO) and used at a final concentration of 10 nM for the *in vitro* experiments according to the manufacturer’s instructions.

### siRNA, shRNA and lentivirus infection

Specific siRNAs and shRNA were designed and synthesized by GenePharma Co., Ltd. (Shanghai, China). The siRNA sequences for CCNE2 were: siR-1: GCAGAUAUGUUCAUGACAA; siR-2: CCAAGUUGAUGCUCUUAAA. The siRNA sequences for CARM1 were: siR-1: GGAUAGAAAUCCCAUUCAA; siR-2: GUGUUUGCUUUGUAAGAAA. The shRNA sequence for CARM1 was: GGATAGAAATCCCATTCAA. In *in vitro* experiments, specific siRNAs were used to knock down CCNE2 or CARM1 expression in PC9 and HCC827 cells. In *in vivo* experiments, shRNA (lentivirus) were used to establish CARM1 stably interfered PC9 cells. To generate stable PC9 cells with CARM1 knockdown, lentivirus were constructed in HEK293T cells with pLKO.1-puro plasmids containing CARM1 shRNA. Lentivirus was obtained 48 h after plasmid transfection using Lipofectamine 2000 (Life Technologies) and 2.5 μg/mL Polybrene (Yeasen; 40804ES76) were mixed to infect PC9 cells. Positive PC9 cells stably expressing CARM1 shRNA were selected by 1 μg/mL puromycin (Beyotime, ST551; China).

### RNA extraction and real-time PCR analysis

Total RNA was isolated from PC9 and HCC827 cells using the TRIzol reagent (Qiagen, German). cDNA was obtained by a Reverse Transcription kit with gDNA Eraser (RR047A; Takara). Quantitative real-time PCR was performed using SYBR green real-time PCR master mix by an Applied Biosystems 7300 Real-time PCR system (Applied Biosystems). The real-time PCR procedures were: 10 min of denaturation at 95°C, followed by 40 cycles of 30 s of denaturation at 95°C, 25 s of annealing at 60°C and 30 s of extension at 72°C, respectively. β-actin message was used to normalize the data. The primer sequences were: β-actin (F): 5’-CACCATTGGCAATGAGCGGTTC-3’; ®: 5’-AGGTCTTTGCGGATGTCCACGT-3’. CARM1 (F): 5’-TTCCAGTCACCACTGT TCGCCA-3’, ®: 5’-CCAGGAGGTTACTGGACTTGGA-3’. CCNE2 (F): CTTACGTCACTGAT GGTGCTTGC; ® CTTGGAGAAAGAGATTTAGCCAGG. The relative mRNA expression was calculated by 2^-ΔΔCt^ method.

### Luciferase reporter assays

The proximal promoter region of human CCNE2 gene was amplified by PCR method and then cloned into the luciferase reporter gene plasmid pGL3.0-Basic. To overexpress CARM1, the full-length CDS region of human CARM1 gene was cloned into the eukaryotic expression vector pcDNA3.1 (+). The fabricated constructs were verified by DNA sequencing. 100 ng, 200 ng, 500 ng and 1000 ng of pcDNA3.1 (+)_CARM1 and vector plasmids were transfected into PC9 and HCC827 cells respectively using Lipofectamine 2000 (Life Technologies) according to the manufacturer’s instructions. The transfected cells were cultured for 48 h under 5% CO_2_ at 37°C before being used in subsequent experiments. Then, cells were washed twice with ice-cold PBS and lysed with RIPA buffer (50 mM Tris, 1 mM EDTA, 150 mM NaCl, 1% Sodium deoxycholate, 0.1% SDS) (P0013B; Beyotime; China). The lysed samples were centrifuged for 15 min at a maximum speed (14000 rpm) and used to determine the luciferase and beta-galactosidase, respectively. The luciferase activity analysis in the PC9 and HCC827 cells was evaluated 48 h post-transfection using the Luciferase Reporter Assay System (Promega, USA) according to the manufacturer’s protocols. Luciferase activity from the pGL3.0-Basic reporter was normalized to beta-galactosidase activity to control for transfection efficiency for each sample. The activity of beta-galactosidase production was measured by a photometric enzyme activity assay by measuring the conversion of ortho-nitrophenyl-beta -D-galactopyranoside.

### Immunoblotting

PC9 and HCC827 cells were washed twice with ice-cold PBS and lysed with RIPA buffer (50 mM Tris, 1 mM EDTA, 150 mM NaCl, 1% Sodium deoxycholate, 0.1% SDS) (P0013B; Beyotime; China). The lysed samples were centrifuged for 15 min at a maximum speed (14000 rpm). The whole cell extracts were quantified by BCA Protein Assay kit (P0012S; Beyotime; China) and boiled for 5 min. Denatured proteins was separated by sodium dodecyl sulfate-polyacrylamide gel electrophoresis (SDS-PAGE) and then transferred to PVDF membranes (Millipore). Subsequently, PVDF membranes were blocked with 5% milk-powder in PBST (1×PBS containing 0.1% Tween-20) for 1 ~ 2 h and then incubated overnight with primary antibodies, including CARM1 (Abcam; ab245467), CCNE2 (Abcam; ab32103), H3R17me2a (Abcam; ab8284), H3R26me2a (Abcam; ab194679), Histone H3 (Abcam; ab1791) and GAPDH (Abcam; ab181602). After washing with PBST, the PVDF membranes were incubated with horseradish peroxidase-conjugated secondary antibody (Sigma). Protein expression was visualized by enhanced chemiluminescence detection kit (Thermo Scientific Pierce). GAPDH was used as a loading control.

### CCK-8 assays

Proliferative activity of PC9 and HCC827 cells was determined by Cell Counting Kit-8 method (CCK-8) according to the manufacturer’ protocols. Cells were cultured in 96-well plates and incubated with 10 μL of WST-8 regent (Dojindo, Japan). After incubation for 2 h at 37°C, OD450 was measured at room temperature.

### Colony formation assays

Colony-formation assays were performed to assess the colony-formation ability of PC9 and HCC827 cells. Cells were seeded into 6-well plates in 2 mL of complete growth medium under 5% CO_2_ at 37°C. Two weeks later, cells were stained with 0.25% crystal violet solution (dissolved in methanol) for 20 ~ 30 min at room temperature. Cell colonies were imaged and counted directly on the plate.

### Immunohistochemistry

Surgically resected fresh NSCLC tissues were formalin-fixed, paraffin-embedded and sectioned. Briefly, the sections were treatment with hydrogen peroxide (H_2_O_2_) and then incubated overnight with the primary antibodies, and then with anti-mouse or anti-rabbit secondary antibody. Lastly, the sections were incubated with 3,3’-diaminobenzidine (DAB) substrate until the positive staining was achieved. The protein expression of CARM1 (Abcam; ab245467) or CCNE2 (Abcam; ab32103) in NSCLC tissues were evaluated blindly by two experienced pathologists. The protein expression was assessed according to staining intensity and percentage of positive cells to generate a histological score (H score). The H score was calculated using the formula: H score = ΣPi (i + 1), where i is the intensity score (0 ~ 3), and Pi is the percentage of stained positive cells (0% ~ 100%).

### Chromatin immunoprecipitation (ChIP)

In brief, PC9 and HCC827 cells were collected and cross-linked with formaldehyde at room temperature for 10 min. Then, the chromatin is randomly sheared by sonication to generate chromatin fragments, generally ranging from 200 to 500 base-pairs. After sonication, 2 μg of CARM1 (Abcam; ab245467), H3R17me2a (Abcam; ab8284), H3R26me2a (Abcam; ab194679) or control rabbit IgG (Beyotime; A7016) were incubated overnight with chromatin fragments and protein G agarose. After washing with low salt, high salt, LiCl and TE buffers, the immune complexes were treated with elution buffer and eluted from the protein G agarose beads. DNA was extracted with phenol/chloroform and followed by ethanol precipitation. Purified DNA was dissolved in TE buffer or ddH_2_O and analyzed by real-time PCR with specific primers for CCNE2 promoter. The primer sequences were as follows: (F) GAAAGACCTGGGTTCCCTGA, ® CTGCAACTCCTGGATTTCGG.

### Mouse xenograft model

About the animal models, a total of 15 BALB/c nude mice (Female; 6 ~ 8 weeks) were were purchased from the Model Animal Research center of Nanjing University (Nanjing, China) and used for the *in vivo* experiments (five mice per group). The mice were randomly divided into three groups (NC, CARM1 KD and CARM1 KD + CCNE2 OE; five mice per group), and 5×10^6^ cells in 0.1 mL PBS (10% Matrigel) were injected subcutaneously in the flank regions of each mouse. The tumor volume was measured every four days using a caliper and calculated with the formula: 0.5 × Length × (Width)^2^. All mice were euthanized by asphyxiation with CO_2_ gas in a semi-closed chamber when a tumor was greater than 0.5 cm^3^ in volume. And then, xenograft tumors were surgically excised, weighted and photographed. The animal experiment was repeated once and all procedures were approved by the Animal Care and Use Committee of Nanjing Medical University.

### Statistical analysis

All statistical analysis was done using GraphPad Prism 5 (GraphPad Software, Inc., California, USA). Statistical significance was determined using Student’s *t*-test. Data were shown as the Mean ± SD (Standard deviation). A *P*-value less than 0.05 was considered to be statistically significant. *P < 0.05, **P < 0.01.

## Supplementary Material

Supplementary Figure 1
