# Supplementary material for: CARM1 promotes non-small cell lung cancer progression through upregulating CCNE2 expression
Source: Aging (Albany NY). 2020 Jun 2;12(11):10578–93. doi: 10.18632/aging.103280 (PMC7346078; doi:10.18632/aging.103280)
Supplement: Supplementary Figure 1 [file aging-12-103280-s001..pdf]

SUPPLEMENTARY FIGURE

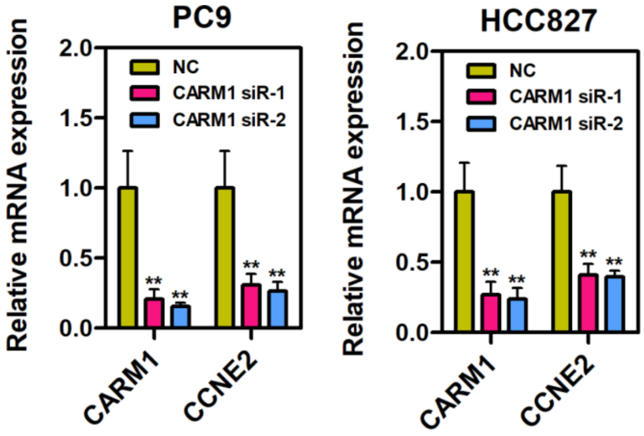

Supplementary Figure 1. The mRNA levels of CCNE2 was downregulated in CARM1-depleted PC9 and HCC827 cells by Real-time PCR assays. GAPDH was used as an internal control. The data were presented as means ± SDs of three independent experiments; $^{**}P < 0.01$.
